# Clay hydroxyl isotopes show an enhanced hydrologic cycle during the Paleocene-Eocene Thermal Maximum

**DOI:** 10.1038/s41467-022-35545-2

**Published:** 2022-12-22

**Authors:** Gregory L. Walters, Simon J. Kemp, Jordon D. Hemingway, David T. Johnston, David A. Hodell

**Affiliations:** 1 Godwin Laboratory for Palaeoclimate Research, Department of Earth Sciences, University of Cambridge, Downing Street, Cambridge, CB2 3EQ UK; 2 British Geological Survey, Environmental Science Centre, Nicker Hill, Keyworth, Nottingham, Nottinghamshire NG12 5GG UK; 3 Department of Earth and Planetary Sciences, Harvard University, 20 Oxford St., Cambridge, MA 02138 USA; 4 Geological Institute, Department of Earth Sciences, ETH Zürich, Sonneggstrasse 5, 8092 Zurich, Switzerland

**Keywords:** Palaeoclimate, Hydrology

## Abstract

The Paleocene-Eocene Thermal Maximum (PETM) was an abrupt global warming event associated with a large injection of carbon into the ocean-atmosphere system, as evidenced by a diagnostic carbon isotope excursion (CIE). Evidence also suggests substantial hydrologic perturbations, but details have been hampered by a lack of appropriate proxies. To address this shortcoming, here we isolate and measure the isotopic composition of hydroxyl groups (OH^−^) in clay minerals from a highly expanded PETM section in the North Sea Basin, together with their bulk oxygen isotope composition. At this location, we show that hydroxyl O- and H-isotopes are less influenced than bulk values by clay compositional changes due to mixing and/or inherited signals and thus better track hydrologic variability. We find that clay OH^−^ hydrogen-isotope values (δ^2^H_OH_) decrease slowly prior to the PETM and then abruptly by ∼8‰ at the CIE onset. Coincident with an increase in relative kaolinite content, this indicates increased rainfall and weathering and implies an enhanced hydrologic cycle response to global warming, particularly during the early stages of the PETM. Subsequently, δ^2^H_OH_ returns to pre-PETM values well before the end of the CIE, suggesting hydrologic changes in the North Sea were short-lived relative to carbon-cycle perturbations.

## Abstract (web summary)

Novel measurements of clay hydroxyl isotopic composition show an enhanced hydrological cycle during a period of intense global warming at the Paleocene-Eocene boundary 55.9 million years ago.

## Introduction

The Paleocene-Eocene Thermal Maximum (PETM) represents an intense global warming event that occurred approximately 55.9 million years ago^1,2^ (for summary, see ref. 3). Whereas the exact cause of the PETM remains uncertain, several diagnostic markers exist in the geologic record, including a prominent carbon isotope excursion (CIE) of ∼3–4‰ at the PETM onset and widespread dissolution of marine carbonates^1,3^. The inferred carbon injection led to average global temperature increases of 4–5 °C^1,4^, oceanographic changes including deoxygenation and acidification leading to mass benthic extinctions^5^ and perturbations in the hydrologic cycle^6^.

The global hydrologic cycle is thought to have changed during the PETM relative to today, but large regional variability likely existed (for summary, see ref. 6). Palynological and biomarker isotope studies suggest sustained increases in terrestrial runoff, both in the tropics (e.g. Venezuela^7^) and in high-latitude regions (e.g. Arctic Spitsbergen^8^, New Zealand^9^, North Sea^10^ and Arctic Ocean^11^). In other regions, sedimentological, palynological and paleosol data imply either increasing aridity (e.g. southern Rocky Mountains^12^) or strong seasonality and extreme rainfall events in the subtropics to lower mid-latitudes (e.g. Spanish Pyrenees^13^, and Normandy^14^). Despite these general trends, large uncertainty persists in our quantitative understanding of the PETM hydrologic cycle and the timing of these changes relative to the CIE.

Improving constraints on the PETM hydrologic cycle is of particular relevance for ongoing and future climate change. Anthropogenic warming is predicted to exceed 2 °C by the end of this century even under the stabilisation (RCP6.0) greenhouse gas scenario^15^. Such warming will likely increase hydrologic-cycle intensity, thus exacerbating environmental stress^16,17^. Current models suggest an enhanced hydrologic cycle with elevated precipitation driven by higher evaporation rates^18,19^, a pattern which may already be emerging. However, while spatial changes in total precipitation amount can be modelled accurately, climate models are not yet precise enough to predict spatial variations in the frequency, seasonality, intensity or type of precipitation^17,20^. Testing whether current climate models can simulate the observed hydrologic responses to temperature during the PETM will aid in assessing future hydrological projections in response to global warming.

Large variations in clay mineralogy are recorded in sediments deposited over the PETM in response to changing climate conditions. A major unresolved observation is a conspicuous increase in kaolinite deposition at many mid- to high-latitude sites (e.g. refs. 21–26). Kaolinite is a clay mineral that is generally assumed to form predominantly by intense weathering in humid tropical climates^27–29^. The origin and significance of PETM kaolinite deposition is controversial because it could result from either: (i) increased chemical weathering and kaolinite formation under warmer climate and elevated year-round precipitation^23,30–32^, or (ii) enhanced mobilisation, physical transport, and exhumation of previously weathered material (e.g. laterite soils) during strong seasonal rainfall events^26,28,33^. Whereas the former interpretation of increased chemical weathering is consistent with a direct negative silicate-weathering feedback to elevated temperatures^34^, the latter of increased physical weathering provides a less direct feedback and may indicate a decoupling between climate and weathering intensity. The two processes are not mutually exclusive and evidence exists that both may have occurred at the time of the PETM in different settings.

**Fig. 1:**
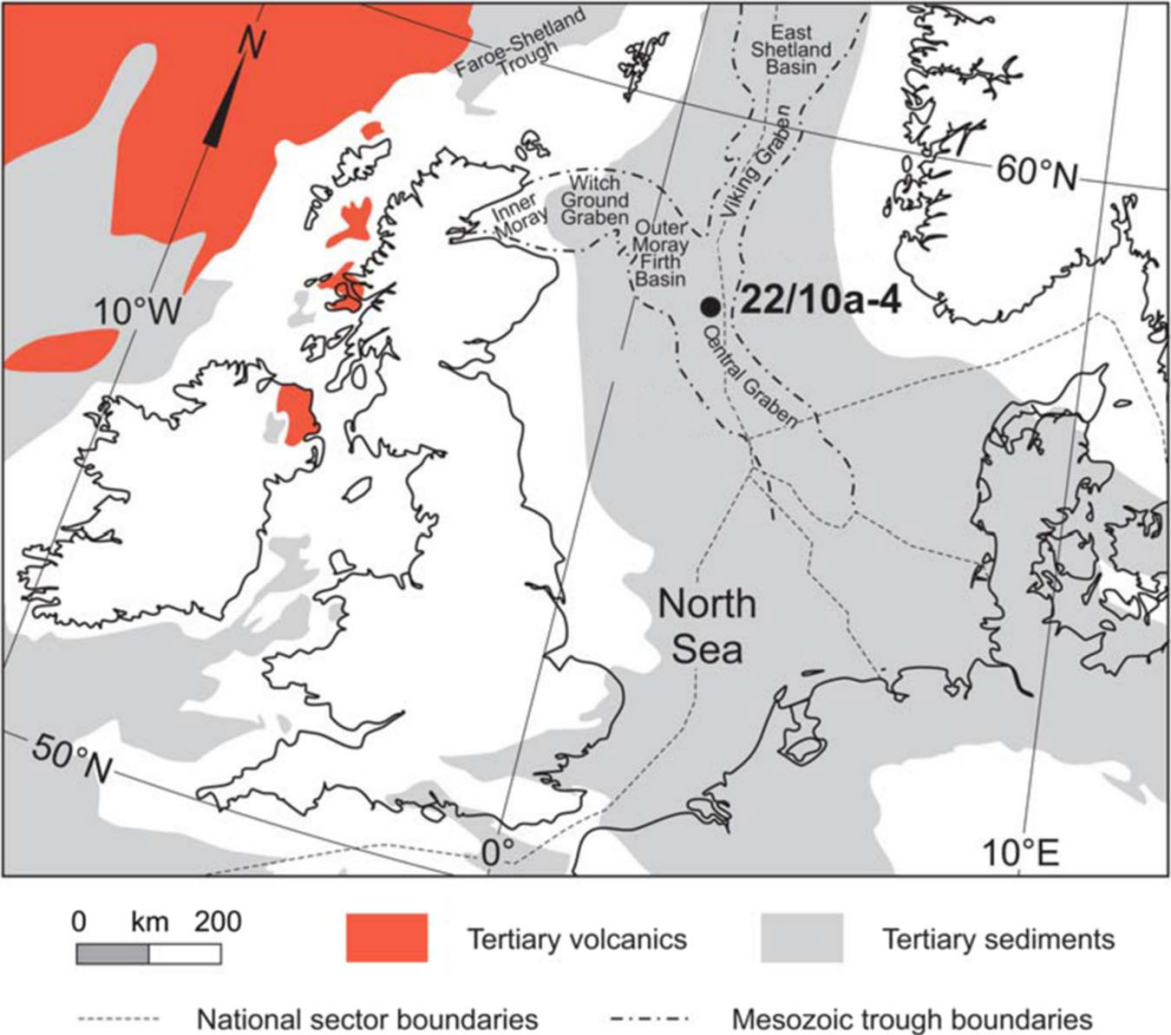
Map of the North Sea Basin, showing the location of well 22/10a-4 and the surrounding geologic features and Paleogene deposits. Figure 1 is reproduced from ref 33. © The Mineralogical Society of Great Britain and Ireland 2016, published by Cambridge University Press, reproduced with permission.

To provide additional constraints on the hydrologic changes across the PETM, we measure the isotopic composition of clay mineral hydroxyl groups from a PETM section in the Sele Formation of the North Sea (well 22/10a-4 (57°44′8.47″N; 1°50′26.59″E; up to ∼500 m paleo-water depth); Fig. 1; refs. 10, 33, 35). During the early Paleogene, the North Sea was a restricted marine basin bounded by Scotland to the north, Greenland to the west and the Fenno-Scandian Shield to the east. The basin contains a highly expanded Paleocene-Eocene transition sequence, uninterrupted but for minor erosion at the base of thin turbidite sandstones^10^. Terrigenous input was high during the Paleogene and thought to be derived primarily from the Scotland Faeroe-Shetland landmass^36^, making this an ideal location to study PETM variability in the hydrologic cycle at a mid-paleo-latitude location (57 °N). Furthermore, by isolating and measuring the hydrogen and oxygen isotope composition of clay hydroxyl groups^37^ (δ^2^H_OH_ and δ^18^O_OH_; Methods) in addition to bulk clay δ^18^O (δ^18^O_bulk_; Methods), our approach aims to avoid potential biases by clay end-member mixing and/or inherited isotopic compositions^26^. We thus expect this record to robustly reflect PETM variability in the hydrologic cycle.

## Results and discussion

**Fig. 2:**
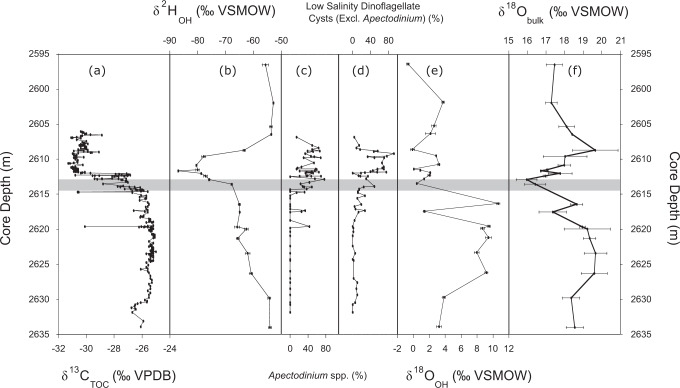
Comparison of the major trends across the onset of the Paleocene Eocene Thermal Maximum (PETM) versus depth in the core from well 22/10a-4. The onset of the Carbon Isotope Excursion (CIE) is shown by the light grey bar. **a** δ^13^C VPDB (Vienna Pee Dee Belemnite) of total organic carbon (TOC)^10^; **b** clay hydroxyl δ^2^H_OH_ VSMOW (Vienna Standard Mean Ocean Water); **c** percentage of the dinoflagellate *Apectodinium* spp. (several species)^10^; **d** percentage of low-salinity dinoflagellate cysts excluding *Apectodinium*^10^; **e** clay hydroxyl δ^18^O_OH_; and **f** the bulk clay δ^18^O_bulk_. Error bars for **b**, **e** and **f** are ±1 σ analytical uncertainty (Methods).

**Fig. 3:**
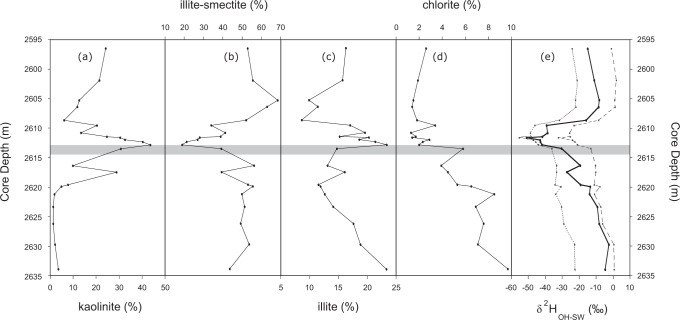
Comparison of the relative percentage of the component clays (note that panels use different scales) and reconstructed clay mineral formation source water versus depth in the core from well 22/10a-4. The onset of the Carbon Isotope Excursion (CIE) is shown by the light grey bar. **a** kaolinite; **b** illite-smectite; **c** illite and **d** chlorite in the <4 μm size fraction from well 22/10a-4^33^ and **e** the reconstructed clay mineral (neo)formation source water δ^2^H (δ^2^H_OH-SW_). Also shown in **e** are end-members assuming 100 % kaolinite (dotted grey line) or 100% illite-smectite (dashed grey line). Reconstructions are calculated using empirical fractionation factors from refs. 39 and 40, respectively (Methods). These end-member values are expected to encompass the range of possible δ^2^H_OH-SW_ values for a given time point.

We compare all clay isotope results relative to the record of bulk organic matter δ^13^C from the same section^10^ and previously reported clay mineralogical data^33^ (Methods; Fig. 2; Fig. 3). The CIE onset in this section is located between 2614.3 and 2613.5 m^10,33^, whereas the post-PETM δ^13^C recovery is not captured in the available δ^13^C data and likely occurs higher in the section.

### Trends in hydroxyl hydrogen and oxygen isotopic composition

Measured δ^2^H_OH_ values decrease gradually by 13.7 ± 0.6‰ over the 15 m leading up to the CIE onset, reaching −67.8 ± 0.3‰ VSMOW (Vienna Standard Mean Ocean Water) at 2613.5 m (Fig. 2b). The CIE onset at 2613.5 m is followed by an abrupt δ^2^H_OH_ decrease of 8.1 ± 0.4‰; δ^2^H_OH_ decreases further throughout the early CIE, culminating in a minimum value of −87.0 ± 0.2‰ VSMOW at 2611.6 m. This δ^2^H_OH_ excursion occurs only between 2613.5 and 2608.7 m, with δ^2^H_OH_ values returning to the pre-PETM baseline at 2606.4 m, well before the end of the CIE (Fig. 2b; ref. 10). In total, δ^2^H_OH_ spans a range of 34.1 ± 0.3‰ (27.6 ± 0.3‰ if the one-point minimum is excluded). Furthermore, the observed δ^2^H_OH_ excursion is coincident with both an increased abundance of low-salinity-tolerant dinoflagellate cysts excluding *Apectodinium*, and of *Apectodinium* itself (a genus abundant during the PETM and possibly associated with enhanced terrestrial runoff^9^; see ref. 38. for discussion) (Fig. 2b, c, d; ref. 10). The strong correlation between dinoflagellate assemblages and δ^2^H_OH_ is particularly apparent between 2612.8 and 2608.7 m, which marks the lowest observed δ^2^H_OH_ values and greatest abundance of low-salinity-tolerant dinoflagellate cysts excluding *Apectodinium* (and of *Apectodinium*).

In contrast to δ^2^H_OH_, δ^18^O_OH_ changes appear to be more closely related to changes in clay composition and δ^13^C (Fig. 2e), but overall correlation with clay oxygen isotope values is weak (e.g. for kaolinite, the strongest correlation, r^2^ = 0.46; *p*-value < 0.01; *n* = 22). At the base of the section, δ^18^O_OH_ averages 3.2 ± 0.3‰ VSMOW but increases to 10.7 ± 0.2‰ VSMOW (omitting a one-point minimum of 1.38 ± 0.13‰ VSMOW at 2617.4 m, which corresponds to a kaolinite content maximum and illite-smectite (I-S) content minimum; Fig. 3a, b). At the CIE onset, δ^18^O_OH_ values again decrease by 10.2 ± 0.2‰ to 0.42 ± 0.04‰ VSMOW and remain stable between −0.7 ± 0.2‰ and 3.7 ± 0.2‰ VSMOW for the remainder of the record.

### Trends in bulk oxygen isotopic composition

Similar to δ^18^O_OH_, bulk clay δ^18^O values are positively correlated with I-S oxygen content (r^2^ = 0.30; *p*-value = 0.01; *n* = 22). Both δ^18^O_OH_ and δ^18^O_bulk_ are also negatively correlated with kaolinite oxygen contribution, with δ^18^O_bulk_ showing a much stronger correlation (r^2^ = 0.83; *p*-value < 0.01; *n* = 22; Figs. 2f, 3b,c) than δ^18^O_OH_. In addition, the magnitude of δ^18^O_OH_ variability (11.3 ± 0.3 ‰) is much greater than that for δ^18^O_bulk_ (3.7 ± 0.8‰ VSMOW; Fig. 2e, f). Correlation between δ^18^O_bulk_ and δ^18^O_OH_ is weak (r^2^ = 0.45; *p*-value < 0.01; *n* = 22). Prior to the PETM, δ^18^O_bulk_ averages 18.6 ± 0.5‰ VSMOW at 2634 m and increases to 19.7 ± 0.6‰ VSMOW at 2623.3 m; however, these high values are not maintained until the CIE onset. Rather, δ^18^O_bulk_ decreases to 17.4 ± 0.7‰ VSMOW between 2621.2 and 2617.4 m, then increases to 18.7 ± 0.3‰ VSMOW at 2616.3 m before decreasing to 16.0 ± 0.6 ‰ VSMOW at the CIE onset at 2612.8 m. Unlike δ^18^O_OH_, δ^18^O_bulk_ begins to return to pre-CIE values after the CIE onset—increasing to 19.6 ± 1.2‰ VSMOW at 2608.7 m—before decreasing to 17.5 ± 0.4‰ VSMOW at the top of the section.

### Estimation of clay hydroxyl source water

Finally, we estimate the hydrogen-isotope composition of clay mineral (neo)formation source water (δ^2^H_OH-SW_) using mineral-specific ^2^H fractionation factors^39,40^ combined with measured δ^2^H_OH_ and clay mineral content trends (Methods; Fig. 3e). Resulting δ^2^H_OH-SW_ closely tracks the measured δ^2^H_OH_ value (r^2^ = 0.90; *p*-value < 0.01; *n* = 22). Absolute values are less negative (ranging from −2.5 to −50.9‰ VSMOW) and exhibit greater variability (48.5‰) relative to δ^2^H_OH_ (34.1 ± 0.3‰), as the most enriched δ^2^H_OH_ values correspond to depths with higher I-S concentrations, while the most depleted δ^2^H_OH_ values correspond to depths with higher kaolinite concentrations, despite kaolinite having a larger fractionation factor.

### Implications of bulk and hydroxyl isotopes on clay origin

The origin of the pervasive increase in PETM kaolinite deposition is debated. Early studies interpreted this trend as reflecting contemporaneous PETM kaolinite formation, indicating increased pedogenesis and weathering under a warm, humid climate with an intensified hydrologic cycle^23,30–32^. More recent studies considered the time required for regolith kaolinitization—with estimates on the order of one million years^28^—and suggested instead that this increase in kaolinite represents increased erosion and exhumation of previously deposited kaolinite that formed well before the PETM^26,33,41^. However, such long timescales refer to the formation of kaolinite from unweathered bedrock but do not necessarily apply to the kaolinization of pre-existing clays in soil profiles. The soil transformation of 2:1-type phyllosilicates (e.g. smectite and illite) into 1:1 types (e.g. kaolinite) has been found to occur on the order of 100 days under laboratory conditions at 150 °C^42^, and significant kaolinization of smectites can occur over a 5–10 ka period under tropical weathering conditions^43^.

Clay oxygen isotopes provide information about possible mineral sources and their genesis. As such, bulk oxygen isotope measurements of PETM-age clays have been analysed previously; for example, from the Bass River section on the New Jersey margin, which similarly contains a mineral assemblage of smectite, illite, kaolinite and quartz^26^. In that study, a strong correlation was observed between smectite and δ^18^O_bulk_ (r^2^ = 0.96) and between kaolinite and δ^18^O_bulk_ (r^2^ = 0.88). This suggested that δ^18^O_bulk_ variability resulted from compositional changes in the proportion of kaolinite and smectite—each with constant δ^18^O_bulk_ values—rather than changes to the δ^18^O_bulk_ of the individual clay minerals across the PETM. Applying a similar analysis to our δ^18^O_bulk_ measurements, we find that only kaolinite shows a similarly strong correlation (r^2^ = 0.83) with δ^18^O_bulk_ (Table 1). This implies that kaolinite across the section has a fairly constant δ^18^O_bulk_ value, suggesting that kaolinite δ^18^O_bulk_ does not necessarily capture isotopic changes across the PETM; this could be the result of kaolinite erosion from a pre-existing source or from kaolinization of existing soil profiles (where δ^18^O_bulk_ will only be partially affected by the addition of hydroxyl groups).

**Table 1:** Correlation coefficients between the amount of oxygen or hydrogen contributed by the component mineral (i.e. mineral relative abundances scaled by structural or hydroxyl weight %; Methods) and the recorded δ^18^O_bulk_, δ^18^O_OH_ or δ^2^H_OH_ (ordinary least squares)

Mineral	r^2^ correlation of isotope contribution		
	δ^18^O_bulk_	δ^18^O_OH_	δ^2^H_OH_
Chlorite	**0.281**	**0.446**	0.157
Illite	**0.194**	0.110	0.005
Illite-smectite	**0.305**	**0.323**	**0.482**
Illite (total)	0.042	**0.340**	**0.511**
Kaolinite	**0.833**	**0.456**	**0.380**
Quartz	**0.483**	—	—
Smectite	**0.460**	**0.248**	**0.187**

Calculations for illite (total) include the illite component of illite-smectite (I-S). Calculations for smectite refer to the smectite component of I-S. Correlations which are significant at a 95% confidence interval are in bold (*p*-value < 0.05).

By contrast, the relative amounts of oxygen contributed by smectite, illite, quartz and chlorite components do not strongly correlate with δ^18^O_bulk_, δ^18^O_OH_ or δ^2^H_OH_, with r^2^ values never exceeding 0.52 (Table 1). Kaolinite content—which contributes up to 75% of the oxygen and hydrogen for the hydroxyl measurements—also shows poor correlation to δ^18^O_OH_ and δ^2^H_OH_, suggesting that the variation in the hydroxyl isotopes over the section is not driven by changes in the clay-sized mineral proportions.

We thus do not observe strong clay compositional effects in the section studied here. In fact, the δ^2^H_OH-SW_ variability increases when measured isotopic values are corrected for clay composition (i.e. by mineral-specific fractionation factors), suggesting a greater change in source water isotopic composition than is actually measured. Furthermore, the measured δ^2^H_OH-SW_ decrease (48.5‰) is consistent with *n*-alkane δ^2^H data from the same region (the Normandy Vasterival section), where a decrease of 60‰ was measured across the early PETM^14^. The observed changes in δ^2^H_OH_ are thus best explained by the formation and alteration of clays during the PETM, rather than by the erosion of pre-existing deposits.

In order for kaolinite formation to capture changing source water isotopic composition across the PETM, it must have formed via a faster mechanism than regolith kaolinization (as regolith kaolinization is estimated to take longer than the duration of the PETM^28^). We therefore suggest that kaolinite was formed by the transformation of 2:1-type phyllosilicate clays (e.g. smectite and illite) into 1:1-type clays (e.g. kaolinite) within soil profiles. This kaolinization process of smectitic precursors leads to the addition of new hydroxyl groups—which will isotopically reflect meteoric water—and has been shown to occur under warm, humid conditions at rates sufficiently fast to capture changes in rainfall isotope composition across the PETM^44,45^.

If, as argued above, clay formation and alteration during the PETM is the major contributor to δ^2^H_OH_ in this section, then the effect of temperature on δ^2^H_OH_ must also be considered, as temperatures in the North Sea region increased over the PETM. Current models for clay hydroxyl group H-isotope fractionation factors (^2H^α_OH_) predict values less than one^39,40^, meaning that clay hydroxyl group H-isotopes are depleted relative to the hydroxyl group source water. Less isotopic fractionation occurs at higher temperatures, so we would therefore expect temperature increases to result in higher δ^2^H_OH_ values over the CIE in contrast to the observed lower values.

The δ^2^H_OH_ signal may be somewhat biased towards marine values by post-depositional isotopic exchange; however, in contrast to exposed terrestrial clays, there is little evidence for hydrogen isotopic exchange in deep-sea buried clays over ∼3 Myr outside of the <0.1 μm fraction^46^, so significant hydroxyl exchange is unlikely. Contamination by organic matter may also affect the accuracy of δ^2^H_OH_ measurements; however, we consider this effect to be relatively minor to the overall δ^2^H_OH_ trend (see the Supplementary Information for a more complete discussion).

### Paleohydrologic implications of δ^2^H_OH_

Taking into account the weak or insignificant correlations between measured isotope values and clay mineral proportions, and the negative relationship between temperature and ^2H^α_OH_, we interpret the hydroxyl isotope record as capturing changes in the isotopic composition of the source water. Although the decrease in δ^2^H_OH_ at the PETM onset is the largest observed change, both the measured δ^2^H_OH_ and the calculated δ^2^H_OH-SW_ show a clear trend towards more negative values in the period leading up to the PETM onset. This trend towards more negative δ^2^H_OH_ values precedes the CIE, suggesting that hydrologic changes during the PETM were not solely a result of changes in the carbon cycle. The pre-PETM decrease in δ^2^H_OH_ may instead reflect increased terrestrial input to the North Sea, as sea level in the basin was lowered by ∼100 m during this time^47^. However, there is additional palynological evidence from a close-by North Sea well, where significant changes to vegetation were documented prior to the CIE, implying the pre-PETM decrease in δ^2^H_OH_ does reflect hydrologic changes which predate the CIE^48^.

The 8‰ VSMOW decrease in δ^2^H_OH_ at the onset of the PETM is interpreted to reflect a decrease in the δ^2^H_OH_ of rainfall. We propose that the mechanism causing this δ^2^H_OH_ decrease is the amount effect^49^—an observed negative correlation between monthly mean rainwater isotope composition and the total amount of precipitation, typically seen in the tropics at low elevation due to increased precipitation intensity in those regions. Increased PETM precipitation amount is further evidenced in this section by the concurrency of the δ^2^H_OH_ excursion and the increased abundances of the low-salinity-tolerant dinoflagellate cysts (and *Apectodinium*), implying that δ^2^H_OH_ accurately reflects the δ^2^H of rainfall during the PETM.

The measured δ^2^H_OH_ decrease during the PETM may reflect high intensity precipitation events where rainfall is depleted in ^18^O and ^2^H; for example, those resulting from tropical cyclone activity^50^. TEX_86_-derived sea-surface temperatures for the North Sea suggest a temperature increase of at least ∼10 °C across the CIE onset with temperatures exceeding 30 °C^51^. Such high temperatures exceed the tropical convective threshold^52^, and may have contributed to the high rainfall intensity. Our δ^2^H_OH_ results are consistent with previous interpretations that global warming during the PETM caused an intensified hydrologic cycle and northward migration of storm tracks^10,11,41^.

The δ^2^H_OH_ values return to their pre-PETM baseline before the CIE termination, suggesting that increased rainfall intensity around the North Sea was short-lived; this too is consistent with prior δ^2^H measurements from the region, where short-lived increases in seasonality have been inferred from *n*-alkane δ^2^H in the Normandy Vasterival section^14^.

Similar hydrological behaviour during the CIE is suggested by mineralogical data from the Fur section in Denmark, where the total clay fraction, clay mineral proportions and the clay chemical index of alteration return to pre-PETM values before the end of the CIE^53^. Lithium isotopic composition measurements from the Fur section similarly show an excursion at the PETM onset before beginning to return to pre-PETM isotopic values during the CIE^54^. However, relative changes in proxies around the CIE onset differ in the Fur section compared to ours—in particular, only the total clay fraction seems to increase before the CIE onset, and kaolinite in this section is mostly present after the CIE onset. The Fur section is less expanded over the start of the PETM (i.e. the Stolleklint clay layer), suggesting that early hydrological changes may not have been fully captured.

Also of note is that the 8 ‰ VSMOW decrease in δ^2^H_OH_ at the PETM onset appears to precede the CIE in this section. This may offer further evidence that hydrologic changes during the PETM were not a result of carbon excursion. However, the CIE onset recorded in this section is complex and potentially influenced by multiple confounding factors, including variable mixing of different carbon sources at time of deposition^55^ (where carbon sources could be either marine or terrestrial, and may or may not reflect newly sequestered carbon), and possible variability caused by a pre-onset carbon isotope excursion, which has been recorded in other sections^56^ (although is not present in this section’s measured record).

### Future Direction

This study uses the recently developed differential thermal isotope analysis (DTIA) method to report clay δ^2^H_OH_ across the PETM^37^. Clay hydroxyl isotopic composition is not a well-developed area of research; future work will benefit from better constraints on hydrogen isotopic fractionation factors for clay hydroxyl groups (^2H^α_OH_). There currently exist a limited number of ^2H^α_OH_ values in the literature^39,40^, and reported values assume that fractionation is not influenced by variations in chemical composition (i.e. the interlayer metal ions present) or mineralogical composition (i.e. the ratio of illite to smectite within illite-smectite). New ^2H^α_OH_ estimates for a suite of clay minerals under environmentally relevant conditions are needed to more accurately correct for changing clay composition and estimates of paleo-precipitation δ^2^H variability. Methodological improvements for the removal of organic matter without alteration of the clay hydroxyl isotope composition, or better constraints for the influence of organic matter on DTIA δ^2^H_OH_ measurements, will also improve the certainty of this proxy. Similar clay isotope measurements should be made in other PETM sections to determine whether the δ^2^H_OH_ values reported here indicate pervasive hydroclimate changes at the PETM onset or were driven by local effects in the North Sea Basin. We emphasise the importance in future studies of carefully evaluating the effects of changing clay composition on the δ^18^O_bulk_, δ^18^O_OH_ and δ^2^H_OH_ before an environmental interpretation is proffered.

In conclusion: we have applied a recently developed method for measuring δ^2^H and δ^18^O of the hydroxyl group in clay minerals to reconstruct past hydrological changes during the PETM. We observe a large decrease in δ^2^H_OH_ coinciding with the CIE, which we interpret to reflect increased precipitation—possibly resulting from increased tropical cyclone activity—in response to global warming during the PETM. Our interpretation is supported by concomitant increases in the abundances of low-salinity dinoflagellates^10^, indicating increased runoff to the North Sea Basin. Hydrologic changes are short-lived relative to the CIE, as evidenced by δ^2^H_OH_ values and low-salinity-tolerant dinoflagellate abundances returning to pre-PETM values before recovery of the carbon cycle. Taken together, these results imply increased kaolinization, silicate-weathering intensity, and hence humidity, in response to warming events in a greenhouse world. Comparing these and future PETM clay δ^2^H_OH_ measurements to predictions from isotopically enabled models offers the promise of quantitative reconstructions of paleohydrologic conditions that can enhance our understanding of hydrology and pedology in a warming world. Moreover, clay hydroxyl group isotopes are a largely untapped proxy, which can greatly improve our understanding of clay provenance and paleoclimate in the past, provided the compositional changes in clay mineralogy are fully characterised.

## Methods

### Isotope notation

All isotope ratios are reported in traditional “delta” notation; for example, written here for ^2^H/^1^H as

1 $${{{{{{\rm{\delta }}}}}}}^{2}{{{\mbox{H}}}}=\left(\frac{{\,}^{2}R_{{{{{\rm{sample}}}}}}}{{\,}^{2}R_{{{{{\rm{standard}}}}}}}-1\right) * 1000{{\textperthousand }},$$

where ^2^*R* is the ^2^H/^1^H ratio and “standard” refers to Vienna Standard Mean Ocean Water (VSMOW) for ${\delta }^{2}{{\mbox{H}}}$ and ${\delta }^{18}{{\mbox{O}}}$ of hydration water and clay bulk oxygen or to Vienna Pee Dee Belemnite (VPDB) for ${\delta }^{13}{{\mbox{C}}}$ for calcite. All results are reported in units of “per mil” (‰).

### Samples

We measured 22 samples in this study consisting of separated <4 μm size fractions of sediment samples across the PETM from the Sele Formation, taken from well 22/10a-4 (57°44′8.47″N; 1°50′26.59″E; up to ∼500 m water depth) located in the central North Sea.

Bulk organic matter δ^13^C has been reported elsewhere for the same section^10^. In the late Paleocene prior to the PETM, δ^13^C remains relatively constant at −25 ± 1‰ VPDB (Fig. 2a). CIE onset in this section is located between 2614.3 and 2613.5 m^10,33^, with a large δ^13^C decrease to −30 ± 1‰ VPDB at 2612.0 m. Carbon isotope values remain low (−30.7 ± 0.3‰) until at least 2605.0 m; post-PETM δ^13^C recovery has not been captured with the available data and likely occurs higher than measured in this section.

Samples of the same <4 μm size fractions were used previously for a clay mineralogical study^33^. The clay mineral assemblage in the section comprises a mixture of kaolinite, illite, illite-smectite (I-S) and chlorite. Illite (13–23 wt.%) and I-S (44–54 wt.%) dominate near the base of the section (2634.0–2620.0 m), with chlorite content also at its highest in this interval (7–10 wt.%; Fig. 3). In contrast, kaolinite concentrations are low during this period (1–4 wt.%), but begin to increase at 2620.0 m and rise irregularly to a maximum of 44 wt.% just after the CIE onset at 2613.5 m; this is mirrored by a decrease in I-S concentration to 19 wt.% (Fig. 3a, b). Following this peak, between 2612.0 and 2608.0 m kaolinite, illite and chlorite concentrations decline to 6–32 wt.%, 9–20 wt.% and 1–3 wt.%, respectively, whereas I-S returns to 27–52 wt.% (mirroring the pattern in kaolinite concentrations). In the upper-most section between 2608.0 and 2596.0 m, I-S again dominates (53–68 wt.%), but illite (10–16 wt.%) and chlorite (1–3 wt.%) are less abundant than in the base of the section (Fig. 3c, d), whereas kaolinite content remains higher (12–24 wt.%) than before the PETM onset.

### Sample preparation and measurement—hydroxyl isotopes

**Fig. 4:**
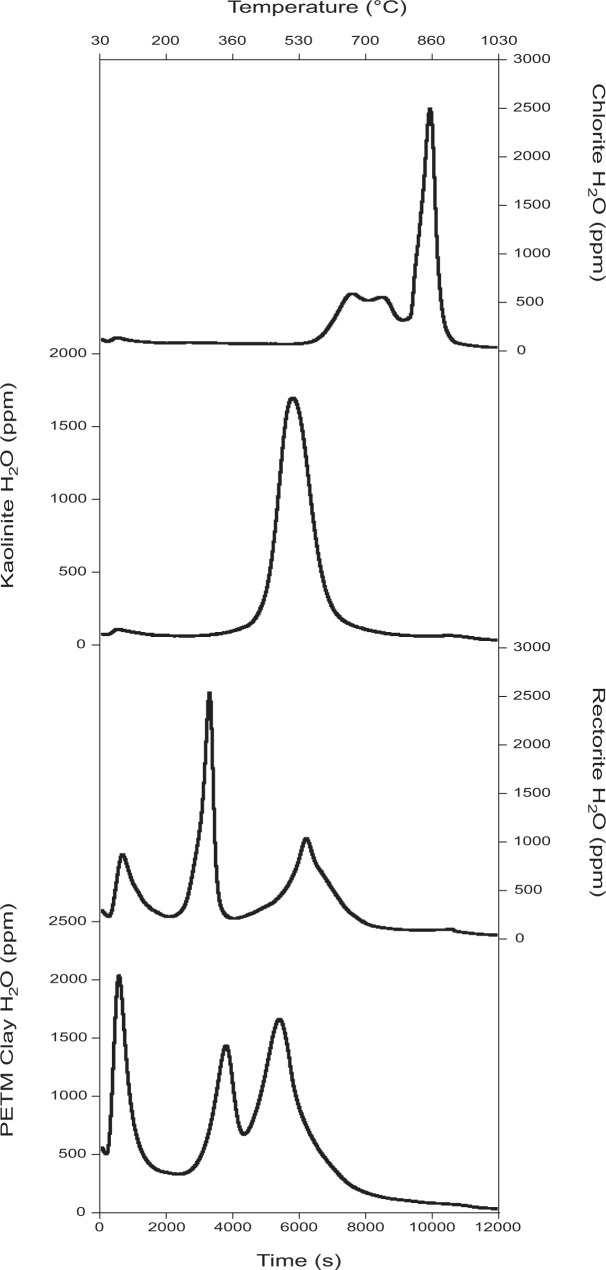
The dehydration profiles of the Paleocene-Eocene Thermal Maximum (PETM) clay assemblage (at 2613.48 m) compared to pure standard samples of clays similar to those found in the assemblage. These dehydration profiles were obtained by using a heating rate of 5 °C per minute between 30 and 1030 °C. No chlorite dehydroxylation peak is seen in our PETM samples, suggesting that the chlorite is sedimentary and undergoes dehydroxylation at lower temperatures^61^. The pure kaolinite sample was provided by IMERYS from Blackpool Pit, St. Austell pluton, Cornwall, UK25. The rectorite standard was purchased from the Clay Minerals Society, originating from Garland County, Arkansas, USA. The chlorite sample is a metamorphic chlorite of unknown origin.

Details of the initial preparation and isolation of the <4 µm samples are given in ref. 33. Before beginning isotope measurements, a test sample was heated using a heating ramp of 5 °C per minute to 1030 °C to identify the temperature range of dehydroxylation (Fig. 4).

**Fig. 5:**
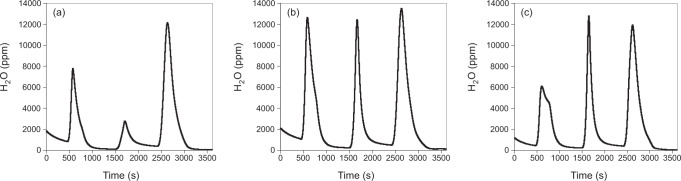
Comparison of the final measured dehydration profiles of three Paleocene-Eocene Thermal Maximum (PETM) samples. The dehydration profiles shown are from depths **a** 2616.34 m, **b** 2613.48 m and **c** 2611.59 m. The first and second peaks, which are measured between 30–250 and 250–390 °C, respectively, likely reflect exchangeable interlayer water. At 250 °C and 390 °C, 10 min isothermal steps were used to increase peak separation. The second peak varies in size across the samples, being between 12 and 51% of the size of the third peak, and thus is too small for accurate isotopic measurements. Including the second peak as part of the hydroxyl peak in isotope analysis leads to only small changes in reported values, and does not change the overall trends in δ^18^O_OH_ and δ^2^H_OH_.

Before making the hydroxyl isotope measurements, we first treated the <4 µm samples with 2 ml of 2 M sodium acetate/acetic acid buffer solution to remove any traces of carbonate. Following acid treatment, the samples were dried overnight at 40 °C and then measured with the Differential Thermal Isotope Analysis (DTIA) system^37^, using a heating programme optimised for maximum separation of the different forms of water contained in the clay minerals (Fig. 5). All heating ramps used were at a 40 °C / min rate to give sharp water peaks. Sample sizes varied between 16 and 31 mg and were adjusted to give a hydroxyl peak height of 11,000–14,000 ppm water in the optical cavity of the Picarro 2130 analyzer. The δ^18^O and δ^2^H were calculated by integrating the H_2_O, δ^18^O and δ^2^H traces for the hydroxyl peak and correcting them for background and calibration of the Picarro cavity ringdown laser spectrometer.

Three to four DTIA measurements were taken at each depth (at 2610.82 m, only two measurements could be taken, due to having limited sample). Errors (1 σ) were calculated from the standard deviation of the measurements at each depth.

### Sample analysis—hydroxyl isotopes

To calculate the fractionation factor corrected δ^2^H values from the hydroxyl group δ^2^H, which should represent the approximate source water hydrogen isotopic composition, a number of assumptions are required. Not all clays have hydroxyl fractionation factors reported in the literature, so we must assume that the hydroxyl water was derived from a mixture of illite-smectite and kaolinite only. We then calculate the proportion of hydroxyl water that each clay mineral would contribute to the total hydroxyl water, by comparing the proportions of each clay in the assemblage to the relative weight percentage of hydroxyl in the clay chemical formulae, assuming a typical clay elemental composition. For illite-smectite, we assume that changes to the percentage composition of illite, K_0.65_Al_2.65_Si_3.35_O_10_(OH)_2_, and montmorillonite, Na_0.1_Ca_0.23_Al_2_Si_4_O_10_(OH)_2_(H_2_O)_11.66_ (assuming the montmorillonite is 36.1 % water by weight), do not affect the fractionation factor, but do change the proportion of hydroxyl H-isotopes contributed. The source water values were then estimated by applying the fractionation factors weighted by the water amounts contributed by each clay:

2 $${{{{{{{\rm{\delta }}}}}}}^{2}{{{{{\rm{H}}}}}}}_{{{{{{\rm{SW}}}}}}}=	{{{{{{{\rm{\delta }}}}}}}^{2}{{{{{\rm{H}}}}}}}_{{{{{{\rm{OH}}}}}}}-\big[1000\,{{{{{\rm{ln}}}}}}\,{{{{{{\rm{\alpha }}}}}}}^{2H}_{{{{{{\rm{kaolinite}}}}}}}\,\times \,{f}_{{{{{{\rm{H}}}}}}}{{{{{{\rm{H}}}}}}}_{2}{{{{{{\rm{O}}}}}}}_{{{{{{\rm{kaolinite}}}}}}}+1000\,{{{{{\rm{ln}}}}}}\,{{{{{{\rm{\alpha }}}}}}}^{2H}_{{{{{{\rm{illite}}}}}}-{{{{{\rm{smectite}}}}}}}\,\\ 	\times \,\left(1-{f}_{{{{{{\rm{H}}}}}}}\right){{{{{{\rm{H}}}}}}}_{2}{{{{{{\rm{O}}}}}}}_{{{{{{\rm{illite}}}}}}-{{{{{\rm{smectite}}}}}}}\big]$$

where:

3 $${f}_{{{{{{\rm{H}}}}}}}=\frac{{\%}_{{{{{{\rm{kaolinite}}}}}}}\,\times \,{\%}_{{{{{{\rm{OH}}}}}}\; {{{{{\rm{in}}}}}}\; {{{{{\rm{kaolinite}}}}}}}}{\left({\%}_{{{{{{\rm{kaolinite}}}}}}}\,\times \,{\%}_{{{{{{\rm{OH}}}}}}\; {{{{{\rm{in}}}}}}\; {{{{{\rm{kaolinite}}}}}}}\,+{\%}_{{{{{{\rm{illiite}}}}}}-{{{{{\rm{smectite}}}}}}}\,\times \,{\%}_{{{{{{\rm{OH}}}}}}\; {{{{{\rm{in}}}}}}\; {{{{{\rm{illite}}}}}}-{{{{{\rm{smectite}}}}}}}\right)}$$

α^2^^H^_mineral_ is the water-mineral hydrogen-isotope fractionation factor, and δ^2^H_SW_ is the estimated source water value for δ^2^H. Hydrogen-isotope fractionation factors at 30 °C are taken from refs. 39, 40. for kaolinite and illite-smectite respectively (Table 2). We also estimate δ^2^H_SW_ assuming compositions of 100% kaolinite and 100% illite-smectite, to account for the spread of possible values for δ^2^H_SW_ in order to estimate the errors for the calculations (where the largest error is caused by not having fractionation factors for an average of ∼25% of the hydroxyl isotope contribution).

**Table 2:** The hydroxyl hydrogen fractionation factors used in this study for kaolinite^39^, and for illite-smectite^40^

Mineral	1000 ln α (Hydroxyl Hydrogen) at 30 °C
Kaolinite	−31.6
Illite-smectite	−54.7

No reliable values for hydroxyl oxygen isotope fractionations in clays exist in the literature. As such, it was not possible to carry out this analysis for hydroxyl oxygen. Fractionation factors for bulk oxygen isotope compositions exist; however, our samples contain considerable amounts of quartz of unknown provenance (up to 31%). For such an analysis to be useful, the quartz would have to be separated from the clay fraction, or the oxygen contribution of the quartz otherwise constrained.

### Sample preparation and measurement—bulk oxygen isotopes

Samples were first treated with 1 M hydrochloric acid (HCl) at a greater than 40:1 liquid to solid ratio for four hours to remove carbonates. This is unsuitable for hydroxyl δD measurements as it leads to hydrogen-isotope exchange, but it does not cause problems for bulk oxygen isotope measurements. Samples were then treated with 30% hydrogen peroxide (H_2_O_2_) for 48 h at room temperature to remove organic matter, again at a greater than 40:1 liquid to solid ratio, and were subsequently centrifuged, decanted and rinsed three times with 18.1 MΩ Milli-Q water to remove residual H_2_O_2_. HCl and H_2_O_2_ treatments are shown to negligibly impact bulk δ^18^O values for a kaolinite standard material, supporting the use of these methods (raw δ^18^O = 21.6 ± 0.6‰, *n* = 2; HCl treated δ^18^O = 22.5 ± 0.5‰, *n* = 2; H_2_O_2_ treated δ^18^O = 20.5 ± 0.7‰, *n* = 9).

Following this, samples were dried overnight at 65 °C in a vacuum oven, loaded into a 21-well laser fluorination tray and dried for a further 72 h at 65 °C in a vacuum oven. This tray was then inserted into a laser fluorination chamber, heated to ∼65 °C under vacuum for ∼6 h, and allowed to cool to room temperature before being reacted with 40 Torr fluorine gas (F_2_) for 15 h to remove any remaining hydration water (“pre-fluorination”). Pre-fluorination product O_2_ was then removed under vacuum. To analyse each sample, the chamber was again charged with 40 Torr F_2_ gas and same material was reacted by use of a 50 W CO_2_ laser (Teledyne) following ref. 57; laser power was initially low to prevent sputtering and was gradually increased to ∼10% until no visible sample residue remained. Fluorinated compounds were then removed by liquid nitrogen, residual F_2_ gas was passivated by KBr salt held at 200 °C, and O_2_ gas was purified by passage through a 3 m long gas chromatography column packed with 5 Å molecular sieve as described in ref. 58.

Analyte O_2_ gas was measured on a ThermoFischer Scientific MAT 253 isotope ratio mass spectrometer (IRMS) operated in dual inlet mode; for each sample, gas was analysed in 4-acquisition blocks of 10 cycles each and averaged. Resulting δ^18^O values were corrected to the 2-point calibration SMOW/SLAP scale following ref. 59. by directly fluorinating and analysing VSMOW-2 and SLAP-2 standard material using a CoF_3_ reactor as described in ref. 60. Uncertainty was taken as the difference between replicate sample aliquots.

## Supplementary information

Supplementary Information

Description of Additional Supplementary Files

Supplementary Dataset S1

Supplementary Datasets 2 and 3

Supplementary Dataset 4

Supplementary Dataset 5

Supplementary Software

## Data Availability

The clay isotope data generated in this study, as well as source data for all figures, are provided in the Supplementary Data.

## Code availability

A Mathematica script which can be used in the analysis of raw Picarro L2130i cavity ringdown spectrometer data is included in the Supplementary Software.
