# Supplementary material for: Clay hydroxyl isotopes show an enhanced hydrologic cycle during the Paleocene-Eocene Thermal Maximum
Source: Nat Commun. 2022 Dec 22;13:7885. doi: 10.1038/s41467-022-35545-2 (PMC9780225; doi:10.1038/s41467-022-35545-2)
Supplement: Supplementary file 1 — Supplementary Information [file 41467_2022_35545_MOESM1_ESM.pdf]

Supplementary Information: Clay hydroxyl isotopes show an enhanced hydrological cycle during the Paleocene-Eocene Thermal Maximum

Gregory L. Walters[1] , Simon J. Kemp[2], Jordon D. Hemingway[3,4], David T. Johnston[3], David A. Hodell[1].

[1]Godwin Laboratory for Palaeoclimate Research, Department of Earth Sciences, University of Cambridge, Downing Street, Cambridge, CB2 3EQ, United Kingdom.
[2]British Geological Survey, Environmental Science Centre, Nicker Hill, Keyworth, Nottingham, Nottinghamshire, NG12 5GG, United Kingdom.
[3]Department of Earth and Planetary Sciences, Harvard University, 20 Oxford St., Cambridge, MA 02138, USA.
[4]Geological Institute, Department of Earth Sciences, ETH Zürich, Sonneggstrasse 5, 8092 Zurich, Switzerland.
Corresponding authors: Gregory L. Walters (gw324@cantab.ac.uk).

Effect of Organic Contamination on DTIA Measurements

Prior study of DTIA measurements of opal has found that minor contamination of water amounts, and minimal contamination of isotope composition, can arise from ~1 % organic contamination[1]. Much stronger contamination effects in isotope composition are seen from the presence of carbonates in samples. When making DTIA measurements at high temperatures (> 600 °C), it is crucial to remove carbonate from the samples; fortunately, non-dolomitic carbonate can be removed without exchange at the clay hydroxyl site. However, there is not yet a process for the removal of organics from clay samples without causing hydrogen isotope exchange. As such, the influence of organic contamination on the North Sea samples should be considered.

Teasing apart the relationship between the amount of organics present in the samples, TOC % (total organic carbon), and the measurements of hydroxyl isotopic composition is difficult in this instance. The amount of organic material present in the section is dependent on the amount of terrestrial input to the basin – where terrestrial input is greater, the amount of organic matter is higher. Increased terrestrial input is caused by increased precipitation amount, and hence the clay isotope composition may appear to change in response to TOC %. It is therefore not possible to distinguish whether a correlation between amount of organic matter and isotopic composition indicates a causal relationship, where the amount of organic matter has a significant impact on the hydroxyl isotopic composition, or is simply a

consequence of the fact that the amount of organic matter and the hydroxyl isotopic composition are both dependent on precipitation intensity.

We have undertaken some analysis to understand the correlation between organic matter and hydroxyl isotopic composition. Although we do not have data from the same depth for all samples, we do have 16 samples for which there is a corresponding TOC % measurement at the same depth used for isotopic analysis [2]. Correlation between the δ^2H_{OH} and TOC % for these samples was found to be significant ($r^2 = 0.7356$). The point of maximum depletion in δ^2H_{OH} also coincides with the highest TOC % (depth 2611.59 m; δ^2H_{OH} -86.97 ‰; TOC 2.778 %; for dehydration profile, see Methods). However, there are also differences in the δ^2H_{OH} and TOC % trends – the elevated TOC % is not maintained after the CIE for as long as the lowest δ^2H_{OH}, and the CIE onset at 2613.5 m does not show much impact on the TOC % aside from the spike at 2611.59 m – almost 2 m deeper than the CIE onset. This contrasts to the large decrease in δ^2H_{OH} at the PETM onset. As a result, we suggest it is unlikely that TOC % is the dominant control of the observed trends seen in δ^2H_{OH} signal.

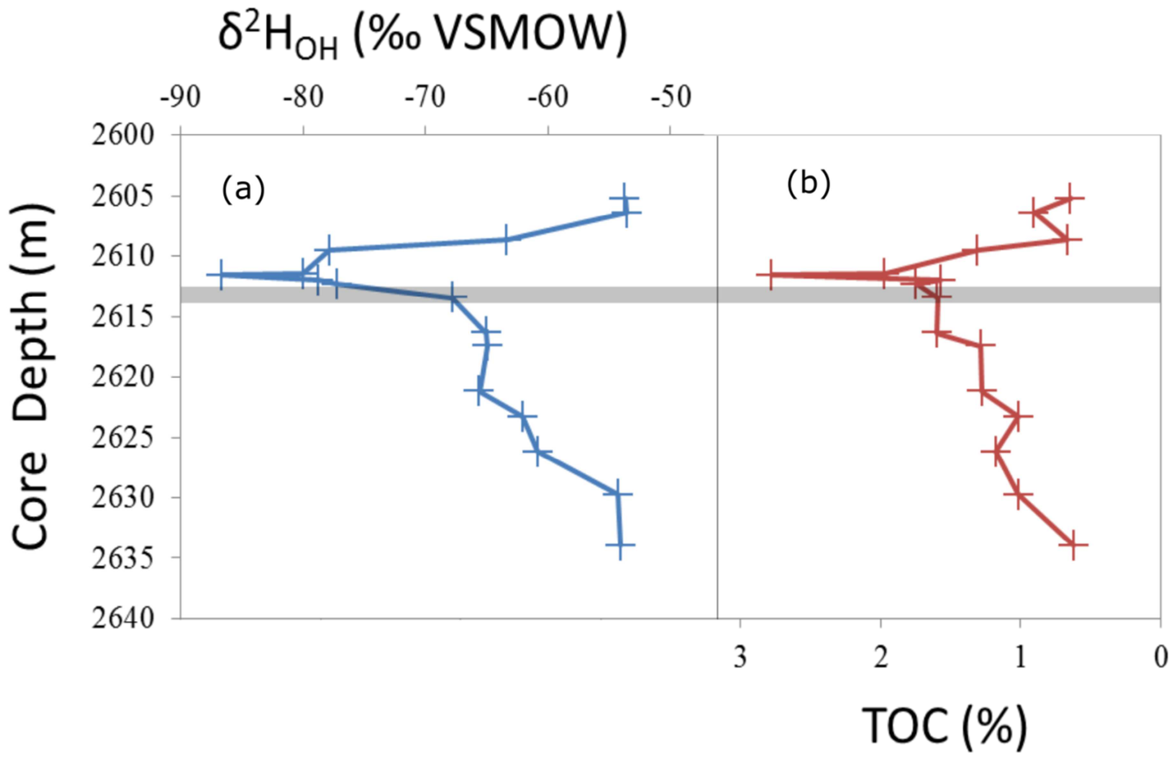

Figure S1. Comparison of paired data for (a) clay hydroxyl δ^2H_{OH} (blue) and (b) total organic carbon (TOC) % (red; data from ref. [2]) across the Paleocene Eocene Thermal Maximum (PETM) onset. The two follow a broadly similar pattern; however, δ^2H_{OH} shows a more rapid response to the Carbon Isotope Excursion (CIE) onset (light grey bar), and takes longer to return to pre-CIE values. This suggests that overall trends in δ^2H_{OH} are not driven by organics present in the samples. The point of maximum δ^2H_{OH} depletion is coincident with the point of maximum TOC % – this may indicate that very high contamination can bias δ^2H_{OH} results, but given other evidence, this likely represents the point of highest terrestrial input to the basin.

We also consider whether there is any significant contribution of organic matter to water peaks by calculating the correlation between the measured water amounts and TOC % across the 16 sample subset. We would expect correlation to be low, as measured water amounts will be strongly related to the proportions of constituent clay minerals, rather than organic content; as such, a high correlation would be surprising (especially as in the original opal measurements, water amount contributions from organics at 1 % contamination were < 10 %[1], and clay minerals are more hydroxyl-rich by weight than opal, meaning that a significantly higher percentage of organic matter would be required to have the same effect).

Indeed, when comparing the water amount of the third peak used for hydroxyl isotope composition measurements to TOC %, we find no correlation ($r^2 = 0.021$), suggesting organic matter contribution to third peak water is insignificant. However, when comparing second peak water amount to TOC %, we find a stronger correlation ($r^2 = 0.507$); this suggests that a significant proportion of organics are removed at lower temperatures than required for clay hydroxyl isotope composition measurement, though confounding factors cannot be ruled out (e.g. if clays are more altered where TOC % is higher, or if clay chemistry is different where TOC % is higher, or some other coincident effect which caused an increase to the size of the second water peak).

In conclusion, it is unlikely that organic contamination is a major factor in these measurements. High amounts of organics may contribute to the one-point minimum seen in δ^2H_{OH}, but there is no evidence at present for large errors in DTIA measurements from non-carbonate contamination, and there is no correlation with the third peak water amounts – while correlation with the second peak water amounts are more significant, and likely indicates that the majority of organics are removed before 390 °C in these samples. Further research into the significance of organic contamination for clay mineral hydroxyl group isotope measurements may be warranted to reduce uncertainty in these measurements.

References

[1] Bauska, T. K., Walters, G., Gázquez, F., & Hodell, D. A. Online Differential Thermal Isotope Analysis of Hydration Water in Minerals by Cavity Ringdown Laser Spectroscopy. *Anal. Chem.*, **90**(1), 752–759 (2017). doi:10.1021/acs.analchem.7b03136

[2] Kender S., *et al.*. Marine and terrestrial environmental changes in NW Europe preceding carbon release at the Paleocene–Eocene transition. *Earth Planet. Sci. Lett.*, **353-354**, 108–120 (2012). doi:10.1016/j.epsl.2012.08.011
