# Supplementary material for: Clay hydroxyl isotopes show an enhanced hydrologic cycle during the Paleocene-Eocene Thermal Maximum
Source: Nat Commun. 2022 Dec 22;13:7885. doi: 10.1038/s41467-022-35545-2 (PMC9780225; doi:10.1038/s41467-022-35545-2)
Supplement: Supplementary file 2 — Description of Additional Supplementary Files [file 41467_2022_35545_MOESM2_ESM.pdf]

Description of Additional Supplementary Files:

Supplementary Datasets 2 and 3: “enhanced PETM hydrology main data figures 2 and 3.csv” – Source data for figures 2 and 3 containing all isotope data from this study as well as clay proportions.

Supplementary Dataset 4: “enhanced PETM hydrology figure 4 data.csv” – Source data for figure 4, containing slow heating ramp water dehydration profile data of three clays and an example PETM clay.

Supplementary Dataset 5: “enhanced PETM hydrology figure 5 data.csv” – Source data for figure 5, containing as measured water dehydration profile data of PETM clays at three depths.

Supplementary Dataset S1: “enhanced PETM hydrology figure s1 data.csv” – Source data for supplement figure 1 containing paired TOC % and δ2HOH data.

Supplementary Software: Zip file containing: “dtia_data_peak_water_isotope_analysis. nb” – Mathematica analysis script for raw Picarro L2130i CRDS data. “Code as text file.txt” – a “.txt” file of the Mathematica analysis script. “HIDS2183-20180205-143903ZDataLog_User.dat” and “HIDS2183-20180205-153909Z-DataLog_User.dat” – example data files to run the Mathematica script against. “example_results.csv” – expected results from running the Mathematica analysis script over the example data. “README.txt” – a readme for the supplementary software package.
